# Tolerance and Inflammation at the Gut Mucosa

**DOI:** 10.1155/2012/738475

**Published:** 2012-06-07

**Authors:** Ana Maria C. Faria, Daniel Mucida, Donna-Marie McCafferty, Noriko M. Tsuji, Valerie Verhasselt

**Affiliations:** ^1^Departamento de Bioquímica e Imunologia, Instituto de Ciências Biológicas, Universidade Federal de Minas Gerais, 31270-901 Belo Horizonte, MG, Brazil; ^2^Laboratory of Mucosal Immunology, The Rockefeller University, New York, NY 10065, USA; ^3^Department of Physiology and Pharmacology, University of Calgary, Calgary, AB, Canada T2N 1N4; ^4^Laboratory for Mucosal Immune Tolerance, Biomedical Research Institute, National Institute of Advanced Industrial Science and Technology (AIST), Yokohama City, Kanagawa 230-0045, Japan; ^5^Tolerance Immunitaire, EA6302, Université de Nice-Sophia Antipolis, Institut National de la Santé et de la Recherche Médicale, 06202 Nice, France

The gut mucosa is the major site of contact with antigens. It holds the largest mass of lymphoid tissue in the body. Under physiological conditions, microbiota and dietary antigens are the natural sources of stimulation for the gut-associated lymphoid tissue (GALT) and for the immune system as a whole. Most GALT cells are activated, and a variety of proinflammatory mediators are found in this site. Regulatory elements, however, counterbalance local inflammatory events in such a way that a delicate yet robust balance keeps the gut homeostasis in check. Normal antigenic contact through the gut mucosa induces two major noninflammatory immune responses, oral tolerance, and production of secretory IgA. However, under pathological circumstances mucosal homeostasis is disturbed resulting in inflammatory conditions such as food hypersensitivity and inflammatory bowel diseases (IBDs). The number of reported cases of food allergy in children grew 18% in the past decade [1]. At the same time, the incidence and prevalence of chronic inflammatory bowel diseases such as ulcerative colitis and Crohn's disease is increasing yearly in US, Europe, Asia, and Latin America [2]. Although therapies for these diseases have improved, they have many side effects and are still only modestly successful for long-term management. The growing incidence of both types of conditions and the need for therapeutic alternatives demands from the scientific community a better understanding of the mechanisms involved in intestinal homeostasis and the pathological settings that may trigger gut inflammation.

 The aim of this special issue is to shed some light on these mechanisms as well as to address alternative therapeutic and preventive approaches for gut inflammatory diseases. It comprises five review articles and ten research articles.

The opening review article by Ramos is a critical appraisal of inflammation as a physiological phenomenon related to the development of multicellular organisms rather than solely a pathological event associated with disease and anti-infectious defense mechanisms. Since the great majority of processes in the gut are a consequence of chronic exposure to large amounts of harmless and often beneficial antigens, the authors discuss how the immune system assimilates these perturbations without generating tissue damage. A. P. da Cunha and H. L. Weiner review the studies on the tolerogenic effects of orally administered anti-CD3 monoclonal antibody in experimental models of autoimmune diseases. Anti-CD3 antibody is biologically active in the gut and may represent a powerful immunologic approach that would be applicable for the treatment of human autoimmune conditions. P. Moingeon and L. Mascarell present a review on the oral lymphoid tissue that comprises various antigen-presenting cells (APCs), resident CD4+ T cells but few mast cells and eosinophils. Interestingly, in the absence of danger signals, the APCs at this site are mostly tolerogenic. These features endow the oral mucosa with properties of a site for immune intervention. Indeed, sublingual vaccines using recombinant allergens are being developed as a novel immunomodulatory strategy to control allergic diseases. The paper by C. T. Murphy and coworkers reviews the role of leukocyte trafficking to the intestinal mucosa in the pathogenesis of inflammatory bowel diseases (IBDs) and the various strategies employed to target leukocyte migration as a putative therapeutic tool to control these disorders.

 In line with these reviewed topics, the original papers in this issue address several aspects of oral tolerance induction, gut inflammatory diseases, and intestinal infection. Two papers present data on the mechanisms involved in oral tolerance to inflammatory reactions occurring in the lung. G. M. Azevedo, Jr., and coworkers showed that intraperitoneal injection of ovalbumin (OVA) in adjuvant minutes before intravenous injection of *Schistosoma mansoni* eggs into OVA tolerant mice blocked the increase of pulmonary granulomas. This indirect effect of oral tolerance to OVA correlates with a reduction in the recruitment of inflammatory cells to the lung suggesting that this is one of the mechanisms by which oral tolerance mediates its indirect effects towards other context-related antigens. The role of regulatory T cells was studied by L. Faustino and coworkers using a model of lung eosinophilic inflammation. Interestingly, at the peak of airway inflammation, the number of regulatory T cells was much higher in allergic mice than in tolerant mice. These regulatory T cells likely play a role in controlling disease progression in allergic mice. The earlier appearance of regulatory T cells in tolerant mice may be the critical parameter that prevents airway inflammation instead of merely limiting it.

 Studying regulatory mechanisms induced by oral tolerance, M. Ruberti and coworkers compared effects in the intestinal mucosa of OVA feeding to BALB/c versus OVA TCR transgenic (DO11.10) mice. OVA-fed BALB/c mice had reduced levels of cytokines in the intestinal mucosa, whereas frequencies of intraepithelial lymphocytes (IELs) expressing Foxp3 and CD103 increased. On the other hand, in OVA-fed DO11.10 mice the intestinal mucosa showed signs of inflammation with increased local cytokine production, and reduction in IEL numbers. Having demonstrated previously that DO11.10 mice could not be rendered tolerant to OVA by the oral route, the authors suggest that the altered proportions of mucosal inflammatory/regulatory T cells and IELs in these mice are related to resistance to oral tolerance induction.

 Two other papers deal with the controversial practice of delaying the introduction of allergenic foods into the infant diet to prevent food allergy development. Contrary to this wide-spread believe, both studies showed that early antigen exposure by breast feeding is able to prevent sensitization in allergy-prone BN rat pups (A. El-Merhibi and coworkers) and in the offspring of allergy-susceptible BALB/c mice (T. Yamamoto and coworkers). Inhibition of specific IgE production and food allergy by oral tolerance to the breast-fed antigen was followed by increases in regulatory T cells and anti-inflammatory cytokines.

 The other reports studied two immunomodulatory strategies for food allergy: a dietary component and a probiotic. O. G. de Matos and coworkers investigated the effect of dietary supplementation with n-3 polyunsaturated fatty acids (PUFA, fish oil source) in an experimental model of food allergy using BALB/c mice. Treatment with n-3 PUFA reduced all inflammatory parameters associated with food allergy including serum levels of antiovalbumin IgE and IgG1, as well as leukocyte recruitment, mucus production, and Paneth cell degranulation in the small intestine. Likewise, administration of *Lactococcus lactis NCC 2287* to BALB/c mice, once sensitized but not before sensitization, reduced allergic manifestations upon allergen challenge. According to A. W. Zuercher and coworkers, the beneficial effect of this strain of *L. lactis* correlates with localized inhibition of Th2 cytokines, particularly IL-13. Therefore, these two strategies characterize promising alternative agents for either prevention or treatment of food allergy.

 In addition to hypersensitivity reactions to food, dysregulated gut immune responses are also observed in IBD. Two other papers address the role of IL-10 and autophagia in IBD development. A. C. Gomes-Santos and coworkers report a kinetic study on the changes in cytokines and lymphoid cells in the intestinal mucosa during the course of spontaneous colitis in IL-10-deficient 129 Sv/Ev mice. Although histological signs of colitis only start at 10 weeks of age reaching overt gut inflammation at 16 weeks of age, decrease in the frequency of CD4+CD25+Foxp3+ regulatory T cells and increase in activated T cells and in IL-17 at the gut mucosa can be detected as early as 6 weeks of age. Interestingly, oral tolerance can be induced in diseased 16-week-old mice by a continuous feeding protocol indicating that this could be an alternative therapeutic strategy for established colitis. Autophagy is another process reported to be involved in intestinal immune homeostasis due to its participation in the digestion of intracellular pathogens and in antigen presentation. To investigate the role of autophagy in the development of experimental models of IBD, N. Wittkopf and coworkers generated mice with intestinal epithelial deletion of the autophagy gene Atg7. Knockout mice showed reduced size of granules and decreased levels of lysozyme in Paneth cells. However, this had no effect on susceptibility in mouse models of experimentally induced colitis leaving this association as an open question for future studies.

 A. Kantele investigated gut immune responses to persistent intestinal infection by monitoring gut-originating plasmablasts in blood. Even in symptomless patients with *Salmonella, Yersinia,* or *Campylobacter* gastroenteritis and in volunteers receiving an oral typhoid vaccine, these persisting pathogens/antigens keep seeding plasmablasts into the circulation. Assaying these cells might provide a less invasive tool for research on intestinal immune responses in diseases in which persisting microbes have a potential pathogenetic significance.

*Ana Maria C. Faria* *Daniel Mucida* *Donna-Marie McCafferty* *Noriko M. Tsuji* *Valerie Verhasselt*
